# Effectiveness of the transitional care model in total knee arthroplasty patients: A randomized controlled trial

**DOI:** 10.1111/ijn.13283

**Published:** 2024-07-11

**Authors:** Ayla Güllü, Betul Tosun

**Affiliations:** ^1^ Faculty of Health Sciences, School of Nursing Hasan Kalyoncu University Gaziantep Turkey; ^2^ Faculty of Health Sciences, School of Nursing Hatay Mustafa Kemal University Antakya Turkey; ^3^ Faculty of Nursing Hacettepe University Ankara Turkey

**Keywords:** functional status, patient readmission, randomized trial, self‐efficacy, total knee arthroplasty, transitional care model

## Abstract

**Aim:**

This study has aimed to assess the effectiveness of the transitional care model (TCM) on functional status, perceived self‐efficacy and healthcare utilization in patients undergoing total knee arthroplasty (TKA).

**Method:**

This randomized controlled study was conducted between February and November 2021 in a public hospital. The study randomly assigned patients to either a 6‐week ‘TCM’ program or usual care. The sample size was *n* = 70, with each group comprising 35 individuals. Patient outcomes, including self‐efficacy, functional status and healthcare service readmission rates, were monitored for TKA patients.

**Results:**

Nursing care based on the ‘TCM’ was found to enhance functional status and increase the level of self‐efficacy among TKA patients, leading to a decrease in healthcare service readmissions.

**Conclusions:**

The study recommends preparing patients and their families for the preoperative and postoperative processes. It emphasizes the importance of providing necessary training and consultancy services under the leadership of orthopaedic nurses responsible for TKA patient care, guided by the principles of TCM.

## Summary statement

What is already known about this topic?

- Treatment for knee osteoarthritis aims to alleviate pain, maintain joint functions and improve the quality of life.
- The success of transitional care relies on effective hospital discharge planning, post‐discharge follow‐up and home support to reduce adverse events.

What this paper adds?

- Patients receiving TCM demonstrated improved outcomes and reduced healthcare service readmission rates.
- Nursing care based on the transitional care model positively affected the functional status of TKA patients.
- TCM increased patients' self‐efficacy levels.

The implications of this paper:

- Patient training and consultancy services should be led by nurses responsible for TKA patients, guided by the principles of TCM.
- TCM should serve as a valuable guide for nurses when planning care for TKA patients.

## 1 INTRODUCTION

Osteoarthritis (OA) is a form of arthritis that typically occurs in older adults and leads to erosion in the articular cartilage, osteophyte formation and subchondral sclerosis. Its main symptoms include pain, temporary morning stiffness, a grating feeling in the joint, instability and a loss of function that can lead to physical disability and impairment in quality of life by placing heavy burdens on individuals. The knee is the most commonly involved joint symptomatically in OA (An et al., 2020; Bilge et al., 2018). The treatment of knee OA aims to relieve pain, eliminate the existing joint range of motion, maintain and improve joint functions, reduce secondary functional failure and improve the quality of life. For this purpose, techniques such as analgesics, nonsteroidal anti‐inflammatory drugs, physical therapy, intra‐articular injections, exercise and patient education are used, and various surgical interventions are performed on patients who do not benefit from these treatments (Çelik et al., 2021).

Total knee arthroplasty (TKA) is a widely accepted treatment when nonsurgical treatment fails and excruciating pain and disability are present (Gaffney et al., 2017). It has been documented that preoperative pain and physical function are the strongest predictors of postoperative pain and physical function. Yet, these alone do not account for the high variability in postoperative outcomes and recovery. It is of great importance to identify interventions that will favourably impact patients' perceptions of surgical outcomes and enhance psychosocial health, and thus, it is essential to identify interventions that can be utilized to improve overall functional outcomes (Hanusch et al., 2014; Magan et al., 2020). The outcomes of TKA surgery provide an improvement in the overall quality of life for many patients. Nevertheless, various complications may develop, depending on the surgery. Thus, a multidisciplinary and continuous approach is essential to achieving successful outcomes in patients after TKA surgery. Preoperative patient management, good surgical technique, well‐managed post‐operative care and discharge training contribute to successful outcomes (Bilik, 2017). In order to eliminate negative consequences for the older adult patient population and to maintain consistency in care between hospital and home, the concept of transitional care as both a research and practice field has emerged in the last few decades (Morkisch et al., 2020). The transition of care occurs when the patient moves from one level of care to another, such as admission to the hospital and being discharged. These transitions are delicate and critical periods when adverse events occur frequently. Transitional care has become a key aspect of patient care in the healthcare system, due to the shortening of hospital stays and the increase in post‐discharge care needs. Transitional care has been described as an array of advanced measures to ensure continuity and coordination of healthcare when the patient is transferred between various healthcare levels (Mardani et al., 2020). The success of transitional care, specifically for older adults, depends on hospital discharge planning, post‐discharge follow‐up and support at home, thereby reducing preventable adverse events such as medication errors, falls and post‐surgical infections. When transitional care is conducted professionally, hospital readmission rates also decrease (Costa et al., 2020). The goals of transitional care are to enhance disease self‐management ability, improve health status and reduce hospital readmission rates through telephone follow‐up, home visits and short message service (Lyu et al., 2021). Previous research with a diverse sample group has determined that the transitional care model is associated with better patient‐reported outcomes and less healthcare use (Allen et al., 2020; Kranker et al., 2018; Liu et al., 2020; Lyu et al., 2021). As a result, many factors, such as the fact that orthopaedic surgery causes more pain than other surgical procedures, the risk of complication development and the long recovery period, require long‐term professional support for individuals who have undergone TKA surgery. Patients should be able to receive counselling easily and quickly, especially after discharge. Therefore, the purpose of this study was to determine the effectiveness of a transitional care model on patients' functional status, perceived self‐efficacy and healthcare utilization in patients with TKA.

## 2 METHODS

### 2.1 Research hypotheses

H1_1_: TKA patients in the TCM group will exhibit a higher level of functional status compared with the control group.
H1_2_: TKA patients in the TCM group will report a higher level of self‐efficacy compared with the control group.
H1_3_: TKA patients in the TCM group will demonstrate lower readmission rates than those in the control group.

### 2.2 Study design

**FIGURE 1:**
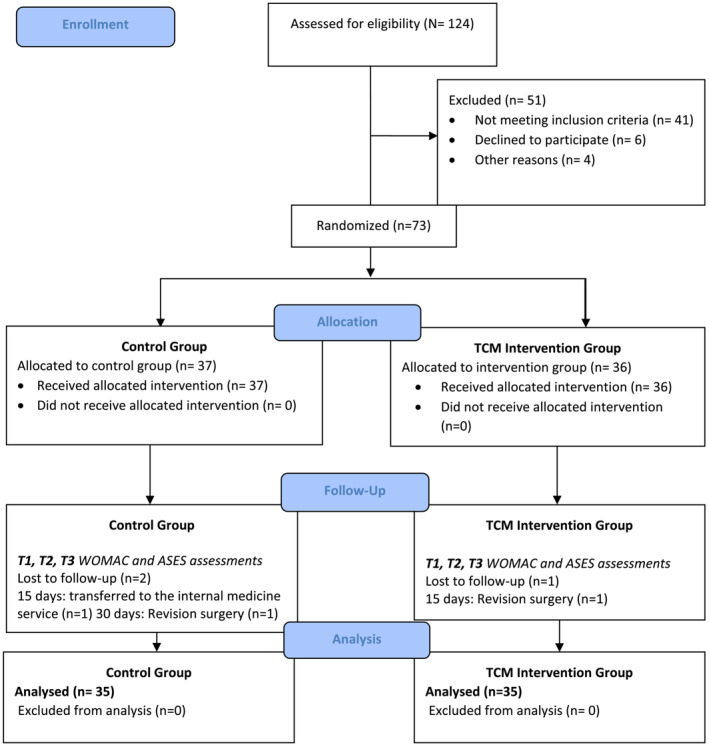
Total CONSORT Statement flow diagram. T1 = baseline, T2 = 2 weeks after discharge, T3 = 9 weeks after discharge. ASES, Arthritis Self‐Efficacy Scale; WOMAC, Western Ontario and McMaster Universities Osteoarthritis Index.

This was a randomized, controlled trial. The study design complies with the CONSORT Statement guidelines (Figure 1). The study was registered at clinicaltrial.gov (NCT05525793).

### 2.3 Participants and study sample

The participants of the study were identified in the Orthopaedics and Traumatology Services of a tertiary healthcare hospital with a total capacity of 50 beds, located in Turkey. The data for the study were collected between February 2021 and November 2021.

The inclusion criteria are as follows:

Who had decided to undergo total knee arthroplasty for the first time due to osteoarthritis,

Volunteered to participate in the research,

Had no psychological, physical or mental disabilities that might hinder communication,

Had no major chronic problems such as end‐stage organ failure, kidney issues, neurological disorders or cancer.

Other inclusion criteria were being discharged home from a hospital, being able to speak Turkish, being available by smartphone and being aged 50 and over.

Exclusion criteria were determined as unwillingness to continue the study and exposure to a disease or trauma that would affect functional independence during the study.

The sample calculation was calculated using the G*Power software, Version 3.1.9.7. The Osteoarthritis Index (Western Ontario and McMaster Universities Osteoarthritis Index [WOMAC]) and Arthritis Self‐Efficacy Scale were considered as primary measurements. When the study of Russell et al. (2011) is taken as a reference (Russell et al., 2011), it was predicted that the *t*‐test would be performed, and with the WOMAC stiffness subscale mean, the difference between the two groups was 1.5 and the standard deviations were 2.43 and 2.32. Moreover, it was predicted that there would be 35 patients for each group (70 patient participants in total) at a 0.68 effect value, 80% power and a 95% confidence interval (CI). When the Liu et al. (2020) study is taken as a reference for the self‐efficacy scale (Liu et al., 2020), the difference in the mean scores of self‐efficacy between the groups was 1.27 points and the standard deviations were 0.82 and 0.84; 20 participants (a total of 40 patient participants) were predicted for each group with an effect size of 0.9, 80% power and 95% CI. In this case, the sample was determined as a total of 70 participants, with 35 patients in each group, based on the larger calculated number of participants.

### 2.4 Data collection instruments

#### 2.4.1 Patient descriptive characteristics form

This form, which was created by the researcher using the literature, consisted of questions to determine some characteristics of the patients, such as age, gender, educational status, marital status, osteoarthritis duration and the presence of other chronic diseases (Doğan et al., 2016).

#### 2.4.2 Western Ontario and McMaster Universities Osteoarthritis Index (WOMAC)

The Osteoarthritis Index is a health status tool that assesses osteoarthritis‐related disability in hip and/or knee osteoarthritis. An array of revisions and changes were made to the WOMAC index, which was first developed in 1982. The latest version is WOMAC 3.1. Items are scored using a 5‐point Likert scale. The Turkish validity and reliability study of the WOMAC OA index was conducted by Tüzün et al. (2005). Cronbach's alpha values for all subscales of the WOMAC index were found to exceed the recommended cut‐off value of 0.70, revealing an acceptable level of reliability for group comparison. It consists of three sections and 24 questions in which pain, stiffness and physical function are questioned. The maximum scores that can be obtained from the index are 20 for the pain subgroup, 8 for stiffness and 68 for physical function. Higher scores indicate an increase in pain and stiffness and an impairment in physical function (Doğan et al., 2016; Tüzün et al., 2005).

#### 2.4.3 Arthritis Self‐Efficacy Scale

(Lorig et al., 1989) developed a scale named ‘Arthritis Self‐efficacy Scale (ASES)’ in order to measure the self‐efficacy perception of patients with arthritis. The validity and reliability study of the scale in our country was conducted by Ünsal and Kaşıkçı (2008). The test–retest reliability of the scale was found to be *r* = .94, the Cronbach's alpha value for internal consistency was .96, and the item total score reliability was found to be between *r* = .59–.96. It consists of 20 statements rated on a 10‐digit visual scale, with a numerical value ranging between ‘Very uncertain = 1’ and ‘Very certain = 10’. It has four sub‐dimensions: self‐efficacy of pain, self‐efficacy of hand‐arm functions, self‐efficacy of foot‐leg functions and self‐efficacy of other symptoms. The minimum score of the scale was determined as 20 and the maximum score as 200, and an increase in the score indicates an increase in the level of self‐efficacy (Ünsal & Kaşıkçı, 2008).

#### 2.4.4 Follow‐up form for readmission to the health services

This form, which was developed by the researcher based on the literature, was prepared to monitor and assess the cases of readmissions to the emergency department or outpatient clinics in the first 12 weeks after discharge (Nall et al., 2020).

### 2.5 Randomization

In accordance with the sampling inclusion criteria, patients were assigned to the TCM intervention and control groups, via the block randomization method. The population of the study consisted of 124 patients for whom the TKA decision was made. The sample size of the study was determined as *n* = 70, each group including 35 individuals. Based on sample size calculations, *n* = 124 patients hospitalized for TKA surgery were evaluated for their eligibility regarding the study. A total of 41 patients were not identified based on the inclusion criteria: 11 had psychological, physical or mental disabilities that might hinder communication; seven had major chronic problems such as end‐stage organ failure, kidney issues, neurological disorders or cancer; seven were unable to speak Turkish; 10 were not available by smartphone; and six were aged 50 years and younger. Patients (*n* = 80) who met the inclusion criteria for the study were informed about the study; six refused to participate in the study, and four were discharged before randomization. A total of two participants (*n* = 2) were excluded from follow‐up due to revision surgery, and one participant (*n* = 1) due to transfer to the internal medicine department. Ultimately, *n* = 70 patients who met the inclusion criteria for participation were given informed consent and enrolled in the study. Patients (*n* = 35) were randomized to the TCM intervention group, whereas (*n* = 35) patients received usual care, and the study was completed with these patients (Figure 1).

### 2.6 Data collection

The clinic team was briefed about the study, ensuring that medical doctors and clinical nurses remained unaware of the group assignments of the patients. The intervention was uniformly administered to all patients by the same experienced nurse researcher, holding a master's degree and possessing 7 years of relevant experience. Initial face‐to‐face interviews took place during hospitalization, wherein participants willing to partake in the study were assessed for eligibility, and information about the study was provided. Upon admission to the service, patients from both groups signed the informed consent form within the first 24 h through face‐to‐face interviews. During this time, the Patient Descriptive Characteristics Form, WOMAC and Arthritis Self‐Efficacy Scale were administered. Follow‐up evaluations occurred at the second, sixth, ninth and twelfth weeks' post‐discharge, utilizing the follow‐up form for repeated hospital applications. Video calls in the second and ninth weeks involved assessments using the WOMAC and Arthritis Self‐Efficacy scales in both groups. Patient readmission data were collected from both hospital records and patient‐reported information.

#### 2.6.1 TCM intervention group

Patients in the TCM intervention group received transitional care plus usual nursing care. Collaboration was established with the patient and their relatives. In accordance with the transitional care model, 6 weeks of counselling and intervention training provided continuity of care and a safe transition, and patients were encouraged about symptom management and a healthy lifestyle.

#### 2.6.2 Control group

The control group received standard nursing care only. Follow‐up evaluations occurred at the second, sixth, ninth and twelfth weeks' post‐discharge using the follow‐up form for readmission. Video calls in the second and ninth weeks included assessments using the WOMAC and Arthritis Self‐Efficacy scales. Patient readmission data were collected from both hospital records and patient‐reported information.

### 2.7 Transitional care program for a patient with total knee arthroplasty

Communication, care coordination, medication reconciliation, functional improvement and self‐management constitute the key components of the transitional care model (Allen et al., 2020; Naylor et al., 2018). In line with the model, during the first face‐to‐face meeting at the hospital, a collaborative approach to care was displayed towards the patients and their relatives in the TCM intervention group. Before the operation, the questions of the patients and their relatives were answered, and information was provided about the preoperative and postoperative processes and medical treatment. After the operation, patients were visited in their wards, and discharge training was delivered. It has been mentioned in the literature that inadequate discharge education leads to the inability of patients to manage their recovery processes on their own. Sharing information with patients promotes an understanding of common goals and expectations (Kang et al., 2019). At this point, education materials prepared by using the views of the service doctors, as well as the nurse in charge of the service and the literature (Holm et al., 2020; NAON: National Association of Orthopaedic Nurses, 2021; Total Knee Replacement ‐ OrthoInfo ‐ AAOS, 2017) were presented to the patients and their caregivers/families. After the patients were discharged, intervention training and counselling were delivered by the researcher for a total of 6 weeks, followed by telephone follow‐up calls, in order to ensure a safe transition and continuity of care. In this application, the participants were reminded of their daily exercises, and they were allowed to continue the exercise program at home. Verbal and documented information about the exercises was shared with the patients and their relatives. Compliance (which medication to receive when and how often, information about the effects and adverse effects of medications and their importance) and continuity with medical treatment were achieved after discharge. Information was given about potential complications and measures to be taken. Lifestyle (weight control, nutrition, prevention of falls, creation of a safe environment, use of walking and assistive devices, significance of exercises, pain management, toilet, bathroom use and daily living activities) training was given after knee arthroplasty. Scheduled control appointments were reminded. Patients were focused towards pain management and self‐care. During this process, the questions of the patients and their relatives were also answered (Table 1).

**TABLE 1:** Content of the transitional care model applied to the intervention group.

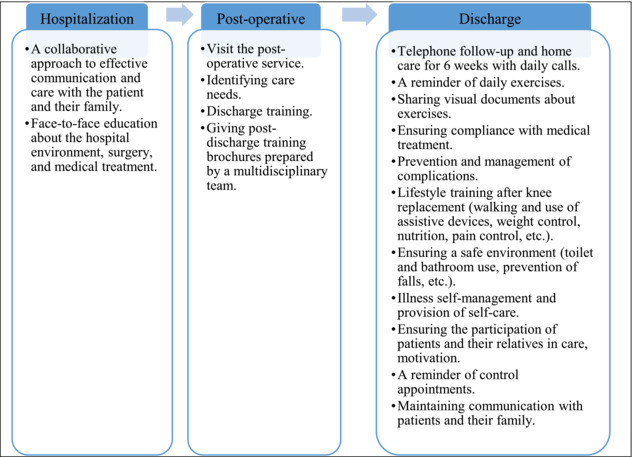

### 2.8 Ethical considerations

Ethical approval was provided by the Hasan Kalyoncu University Faculty of Health Sciences Non‐Interventional Clinical Research Ethics Committee (Date: 19.01.2021, Number: 2021/004). The aim of the study was explained to volunteering patients, who were asked to provide written informed consent. The Helsinki Declaration's principles were applied in all phases of the study.

### 2.9 Data analysis

Data were analysed via the software of IBM SPSS Statistics for Windows, Version 23.0 (IBM Corp., Armonk, NY, USA). Measurement data were expressed as numbers, percents (%) and mean ± standard deviation. Whether the data fit the normal distribution or not was analysed with the Shapiro–Wilk test. Comparison of scores between groups, independent samples *t*‐test for repeated measurements and ratios were compared using Chi‐square or Fisher's exact test. A repeated measures ANOVA test was used to evaluate repeated measures between groups. *p* ≤ .05 was considered statistically significant. Randomization was blinded to outcome assessors who analysed the data.

## 3 RESULTS

### 3.1 Descriptive characteristics of the patients with TKA

More than half of the participants (*n* = 37, 59.2%) were 60–69 years old, 90% were female, and most of them (70%) were middle school graduates. At the onset of the study, no significant difference was found between the study groups in terms of descriptive characteristics, including gender, age, education and duration of osteoarthritis (Table 2).

**TABLE 2:** Comparison of the descriptive characteristics of the participants categorized according to the study group (*n* = 70).

Variable	Total (*n* = 70) *n* (%)	Control (*n* = 35) *n* (%)	Intervention (*n* = 35) *n* (%)	*x* ^2^/*t*	*p*
Age					
50–59	19 (27.1)	9 (12.9)	10 (14.3)	0.365	0.833
60–69	37 (59.2)	18 (25.7)	19 (27.1)		
≥70	14 (20.0)	8 (11.4)	6 (8.6)		
Gender					
Male	7 (10.0)	3 (4.3)	4 (5.7)	0.159	1.000
Female	63 (90.0)	32 (45.7)	31 (44.3)		
Educational status					
İlliterate	18 (25.7)	11 (15.7)	7 (10.0)	1.732	0.421
Middle school graduate	49 (70.0)	22 (31.4)	27 (38.6)		
University	3 (4.3)	2 (2.9)	1 (1.4)		
Marital status					
Married	56 (80.0)	29 (41.4)	27 (38.6)	0.357	0.557
Single	14 (20.0)	6 (8.6)	8 (11.4)		
Employment status					
Unemployed	63 (90.0)	33 (47.1)	30 (42.9)	1.429	0.428
Employed	7 (10.0)	2 (2.9)	5 (7.1)		
Person living with					
Alone	6 (8.6)	2 (2.9)	4 (5.7)	0.729	0.673
Family	64 (91.4)	33 (47.1)	31 (44.3)		
Income level					
Income less than expenses	31 (44.3)	14 (20.0)	17 (24.3)	0.521	0.470
Income equals expense	39 (55.7)	21 (30.0)	18 (25.7)		
Comorbidity					
Yes	44 (62.9)	20 (28.6)	24 (34.3)	0.979	0.322
No	26 (37.1)	15 (21.4)	11 (15.7)		
Osteoarthritis duration					
0–5 years	18 (25.7)	7 (10.0)	11 (15.7)	4.007	0.261
6–10 years	34 (48.6)	18 (25.7)	16 (22.9)		
11–20 years	16 (22.9)	10 (14.3)	6 (8.6)		
≥21 years	2 (2.9)	0 (0.0)	2 (2.9)		
BMI ± SD	32.5 ± 5.25	32.1 ± 4.57	32.8 ± 5.89	−0.566	0.573

*Note*: *x* ^2^ = Chi‐square or Fisher exact test.

Abbreviations: BMI, body mass index; SD, standard deviation; *t*, Student *t*‐test.

### 3.2 Comparison of the WOMAC scores between intervention and control group patients

**FIGURE 2:**
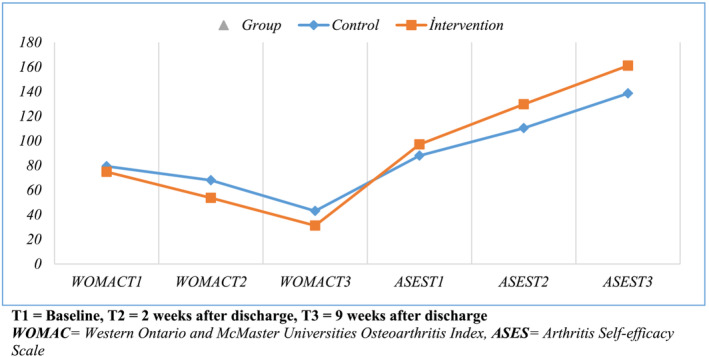
Change of measurement values of WOMAC and ASES scores of groups over time.

The WOMAC total score distributions of the intervention group measured at T1, T2 and T3 were 74.77 ± 17.31, 53.60 ± 17.16 and 31.09 ± 17.10, respectively. Meanwhile, the WOMAC total score distributions of the control group were 79.31 ± 13.95, 67.97 ± 13.34 and 42.97 ± 15.53, respectively. Accordingly, it can be stated that the final WOMAC scores have decreased. It is seen that baseline data are not significant for both groups (*p* = 0.231). Statistically significant differences between the groups occurred at T2 (*p* < 0.001) and T3 (*p* = 0.003) times. The results revealed significant differences in interaction between groups and within groups. Accordingly, it was determined that the group effect (*F* = 13.96, *p* < 0.001) and the main time‐dependent effect (*F* = 160.12, *p* < 0.001) were statistically significant (*F* = 2.58, *p* = 0.086; Table 3 and Figure 2).

**TABLE 3:** Comparison of WOMAC and ASES scores of TCM intervention and control groups.

	T1	T2	T3	Between‐groups	Within‐group	Group*Time interaction
	Mean (SD)	Mean (SD)	Mean (SD)			
WOMAC						
Intervention	74.77 (17.31)	53.60 (17.16)	31.09 (17.10)	*F* = 13.96	*F* = 160.12	*F* = 2.58
Control	79.31 (13.95)	67.97 (13.34)	42.97 (15.53)	*p* < 0.001	*p* < 0.001	*p* = 0.086
*t‐test (p)*	1.209 (0.231)	3.911 (<0.001)	3.044 (0.003)	*np* ^2^ = 0.17	*np* ^2^ = 0.70	*np* ^2^ = 0.03
ASES						
Intervention	97.09 (27.74)	129.69 (16.11)	161.00 (12.79)	*F* = 16.77	*F* = 436.26	*F* = 6.46
Control	87.91 (23.34)	110.29 (19.49)	138.51 (14.90)	*p* < 0.001	*p* < 0.001	*p* = 0.005
*t‐test (p)*	−1.497 (0.139)	−4.539 (<0.001)	−6.774 (<0.001)	*np* ^2^ = 0.19	*np* ^2^ = 0.86	*np* ^2^ = 0.08

*Note*: T1 = baseline, T2 = 2 weeks after discharge, T3 = 9 weeks after discharge, ANOVA at repeated measures →, independent sample *t*‐tests ↓.

Abbreviations: ASES, Arthritis Self‐efficacy Scale; *np* ^2^, partial eta squared (effective power); WOMAC, Western Ontario and McMaster Universities Osteoarthritis Index.

### 3.3 Comparison of ASES scores between intervention and control group patients

When the ASES scores of the groups were examined, it was noticed that the total score distributions of the intervention group measured in T1, T2 and T3 were 97.09 ± 27.74, 129.69 ± 16.11 and 161.00 ± 12.79, respectively. Meanwhile, the ASES scores of the control group were found to be 87.91 ± 23.34, 110.29 ± 19.49 and 138.51 ± 14.90, respectively. Based on this result, it can be stated that ASES final scores have increased. It is seen that baseline data are not significant for both groups (*p* = 0.139). Statistically significant differences occurred in T2 (*p* < 0.001) and T3 (*p* < 0.001) time measurements. Regarding ASES total scores, the main time‐dependent effect (*F* = 436.26, *p* < 0.001), group effect (*F* = 16.77, *p* < 0.001) and time‐ and group‐dependent joint effect (*F* = 6.46, *p* = 0.005) were found to be significant (Table 3 and Figure 2).

### 3.4 The comparison of the WOMAC and ASES sub‐dimension scores between/within intervention and control group patients

When the WOMAC sub‐dimension mean scores were examined, it was seen that baseline data for pain, stiffness and physical function sub‐dimensions were not significant for both groups (*p* > 0.05). A significant difference was found between the patients in the intervention and control groups, regarding pain and physical function sub‐dimension mean scores at T2 and T3 times (*p* < 0.05). When the sub‐dimension scores were analysed according to repeated measurements, a significant difference was found between and within the group in pain (between groups: *p* < 0.001, within groups: *p* < 0.001), stiffness (between groups: *p* = 0.023, within groups: *p* < 0.001) and physical function (between groups: *p* < 0.001, within groups: *p* < 0.001) dimensions, respectively (*p* < 0.05). However, no significant difference was found in group*time interaction in the pain, stiffness and physical function sub‐dimensions (*p* > 0.05; Table 4).

**TABLE 4:** Distribution of WOMAC and ASES sub‐dimensions mean scores after discharge of TCM intervention and control group patients.

	T1	T2	T3	Between‐groups	Within‐group	Group*time interaction
*WOMAC*	Mean (SD)	Mean (SD)	Mean (SD)			
Pain						
Intervention	3.53 (0.52)	2.42 (0.72)	1.41 (0.69)	*F* = 14.14	*F* = 204.91	*F* = 3.97
Control	3.60 (0.50)	2.98 (0.54)	1.90 (0.66)	*p* < 0.001	*p* < 0.001	*p* = 0.024
*t‐test (p)*	0.556 (0.580)	3.650 (0.001)	3.021 (0.004)	*np* ^2^ = 0.17	*np* ^2^ = 0.75	*np* ^2^ = 0.05
Stiffness						
Intervention	3.42 (0.57)	2.31 (0.56)	1.27 (0.71)	*F* = 5.43	*F* = 174.69	*F* = 1.63
Control	3.55 (1.05)	2.84 (0.92)	1.57 (0.74)	*p* = 0.023	*p* < 0.001	*p* = 0.203
*t‐test (p)*	0.631 (0.531)	2.886 (0.006)	1.719 (0.090)	*np* ^2^ = 0.07	*np* ^2^ = 0.72	*np* ^2^ = 0.02
Physical function						
Intervention	2.95 (0.83)	2.16 (0.74)	1.26 (0.74)	*F* = 12.51	*F* = 119.00	*F* = 1.96
Control	3.18 (0.72)	2.78 (0.61)	1.78 (0.67)	*p* < 0.001	*p* < 0.001	*p* = 0.149
*t‐test (p)*	1.241 (0.219)	3.772 (0.000)	3.039 (0.003)	*np* ^2^ = 0.15	*np* ^2^ = 0.63	*np* ^2^ = 0.02
*ASES*						
Pain						
Intervention	4.20 (1.46)	5.77 (1.09)	7.17 (0.57)	*F* = 33.17	*F* = 354.33	*F* = 2.45
Control	3.29 (0.74)	4.42 (1.11)	5.91 (0.66)	*p* < 0.001	*p* < 0.001	*p* = 0.089
*t‐test (p)*	−3.279 (0.002)	−5.105 (0.000)	−8.478 (0.000)	*np* ^2^ = 0.32	*np* ^2^ = 0.83	*np* ^2^ = 0.03
Foot‐leg						
Intervention	3.02 (1.81)	5.09 (1.27)	7.21 (1.01)	*F* = 8.63	*F* = 238.26	*F* = 1.48
Control	2.65 (1.36)	4.17 (1.47)	6.27 (0.96)	*p* = 0.004	*p* < 0.001	*p* = 0.232
*t‐test (p)*	−0.969 (0.336)	−2.643 (0.010)	−3.944 (0.000)	*np* ^2^ = 0.11	*np* ^2^ = 0.77	*np* ^2^ = 0.02
Hand‐arm						
Intervention	8.43 (1.94)	8.89 (1.51)	9.04 (1.36)	*F* = 0.49	*F* = 34.91	*F* = 0.50
Control	8.24 (1.71)	8.65 (1.34)	8.72 (1.14)	*p* = 0.483	*p* < 0.001	*p* = 0.509
*t‐test (p)*	−0.430 (0.668)	−0.703 (0.485)	−1.079 (0.284)	*np* ^2^ = 0.00	*np* ^2^ = 0.33	*np* ^2^ = 0.00
Other symptoms						
Intervention	3.63 (2.30)	6.02 (1.21)	8.50 (0.61)	*F* = 11.97	*F* = 268.87	*F* = 8.31
Control	3.27 (2.27)	4.69 (1.57)	6.70 (1.26)	*p* = 0.001	*p* < 0.001	*p* = 0.002
*t‐test (p)*	−0.670 (0.505)	−3.959 (0.000)	−7.562 (0.000)	*np* ^2^ = 0.15	*np* ^2^ = 0.79	*np* ^2^ = 0.10

*Note*: T1 = baseline, T2 = 2 weeks after discharge, T3 = 9 weeks after discharge, ANOVA at repeated measures →, independent sample *t*‐tests ↓.

Abbreviations: ASES, Arthritis Self‐efficacy Scale; *np* ^2^, partial eta squared (effective power); WOMAC, Western Ontario and McMaster Universities Osteoarthritis Index.

No significant difference was found between the baseline data for the foot‐leg, hand‐arm and other symptoms sub‐dimensions for both groups (*p* > 0.05). A significant difference was determined between the groups in the mean scores of pains, foot‐leg and other symptoms sub‐dimensions at T2 and T3 times (*p* < 0.05). When the sub‐dimension scores were examined with repeated measurements, significant differences were found between and within the group in the dimensions of pain (between groups: *p* < 0.001, within groups: *p* < 0.001), foot‐leg (between groups: *p* = 0.004, within groups: *p* < 0.001) and other symptoms (between groups: *p* = 0.001, within groups: *p* < 0.001), respectively (*p* < 0.05). It was revealed that the group*time joint effect of three sub‐dimensions of pain, foot‐leg and hand‐arm was not significant (*p* > 0.05). It was observed that there was a group*time interaction in the other symptoms (*p* = 0.002) sub‐dimension (*p* < 0.05; Table 4).

### 3.5 Comparison of the rates of hospital readmission between intervention and control group patients

When the admission rates of the groups in four different time periods were analysed, the rate of admission to the hospital (0.14 ± 0.35) of the patients in the control group at time T2 was found to be significantly higher compared to the intervention group (0.00 ± 0.00; *p* = 0.023; Table 5).

**TABLE 5:** Comparison of the rates of readmission to the hospital except for routine controls.

Readmission to hospital (emergency visits/outpatient visits)	T1	T2	T3	T4
	Mean (SD)	Mean (SD)	Mean (SD)	Mean (SD)
Intervention	0.08 (0.28)	0.00 (0.00)	0.00 (0.00)	0.00 (0.00)
Control	0.11 (0.32)	0.14 (0.35)	0.08 (0.28)	0.02 (0.16)
*t* (*df*)	*t* (68) = 0.393	*t* (68) = 2.380	*t* (68) = 1.785	*t* (68) = 1.000
***p***	*0.695*	***0.023***	*0.083*	*0.324*

*Note*: T1 = 2nd week after discharge, T2 = 6th week after discharge, T3 = 9th week after discharge and T4 = 12th week after discharge.

The reasons for admission to the hospital were found to be pain, swelling, hematoma, constipation, wound infection, falling, development of pain in the other leg, systemic diseases and the feeling that the prosthesis does not fit.

## 4 DISCUSSION

In this study, it was revealed that the nursing care given according to TCM had an impact on functional status, self‐efficacy and health service utilization in patients who underwent TKA. At the onset of the study, patients' descriptive characteristics, health conditions, WOMAC scores and ASES scores were similar; thus, the groups were homogeneous at the beginning of the study. This is an indication that both groups were similar at the beginning and that neither group was favoured or opposed, and it is similar in structure to other studies (Lyu et al., 2021; Sun et al., 2020). One of the distinct clinical signs of symptomatic knee OA is the functional limitations experienced by individuals. This situation affects the quality of life in individuals and, thus, results in compromising their activities in their daily lives (Korkmaz et al., 2022). The functional status measurement scores of the intervention group participants at the second and ninth weeks after discharge were significantly lower than the control group. The results revealed that there were significant positive differences in the measurements between and within the group, depending on the time. In a previous study by Magan et al. (2020), it was found that a multimodal therapy program, which includes training on post‐TKA, safety, disease information, exercise and management of complications such as swelling and pain, significantly improved the WOMAC scores of patients (Magan et al., 2020). This result demonstrated that a significant improvement occurred in the functional levels of all patients over time and that the 6‐week follow‐up, counselling and training application provided via a TCM was an effective intervention in improving the functional status of the patients.

Self‐efficacy in arthritis can be enhanced by education and psychological intervention, with the developments in this cognitive dimension in parallel with the developments in health status. It has been reported in the literature that low self‐efficacy in managing pain and distress contributes to the occurrence of depression and is associated with poorer physical functionality (Pinto et al., 2015). In this study, the measurements in all weekly periods in both study groups were determined to be varying, and ASES follow‐up scores increased over time. This result showed that all patients' self‐efficacy levels improved significantly over time. The results of the study suggested that, compared with usual care, transitional care was more effective in increasing patients' motivation and coping with disease symptoms, improving patients' beliefs about arthritis and enhancing their perception of disease self‐management. An increase in the individual's perception of self‐efficacy helps him/her show positive health behaviours (Doğan et al., 2016).

In this study, the rate of readmission (emergency visits and/or outpatient visits) to the hospital in the control group after discharge was found to be higher compared with the intervention group, and a significant difference was observed between the groups in the 6th‐week measurement. In this study, reasons such as pain, swelling, hematoma, constipation, wound infection, falling, pain development in the other leg and the feeling that the prosthesis did not fit were among the reasons for the participants to apply to the hospital. Likewise, it has been suggested in the literature that transitional care decreases the rate of healthcare service utilization, could be an appropriate approach to reduce healthcare utilization costs and can promote sustainability in healthcare systems (Kranker et al., 2018; Li et al., 2022; Ohuabunwa et al., 2021; Patel et al., 2021). In the study, it was considered a very pleasing and positive development that the patients in the intervention group reduced their readmissions to the hospital, thanks to transitional care. However, further studies are needed.

### 4.1 Limitations

This study is subject to several limitations, primarily stemming from the impact of the COVID‐19 pandemic on the execution of transitional care interventions within the hospital setting. The researcher, mandated to implement precautionary measures hindering effective communication, may not have adequately addressed the necessity for transparent communication and addressing patient concerns. Furthermore, post‐discharge follow‐up was conducted through virtual meetings due to the pandemic‐related constraints on in‐person interactions.

Another limitation lies in the absence of a detailed cost analysis of medical records, preventing an assessment of how the transitional care model services may influence hospital costs. It is important to note that the research findings are reliant on patient statements, thus introducing a potential bias since patients may have provided responses influenced by their expectations or perceptions. This scenario underscores one of the challenges encountered during the research implementation, where patients may have responded in a manner deemed desirable by the researcher. Despite these limitations, the study contributes valuable insights within the context of the constraints imposed by the pandemic and underscores the need for future research endeavours to address these challenges more comprehensively.

## 5 CONCLUSION

The study's findings corroborate those of other earlier research that found that applying the transitional care model improved patient outcomes and reduced healthcare service consumption rates. In the study, it was found that the nursing care delivered based on the ‘Transitional Care Model’ improved the functional status of patients who underwent TKA, increased the level of self‐efficacy of the patients and decreased the rate of healthcare service utilization. The TCM intervention applied in TKA patients resulted in further improvement in patients' outcomes. In light of the study results, it is recommended that the patient and the family be prepared for the preoperative and postoperative processes; moreover, the training and consultancy services needed by the patients should be provided under the leadership of the orthopaedic nurse who will care for TKA patients under the guidance of TCM and to use TCM as a guide for nurses when planning nursing care for TKA patients. Further studies can be conducted to analyse the detailed medical records of TKA patients, exploring how TCM impacts hospital costs.

## AUTHORSHIP STATEMENT

Ayla Güllü and Betul Tosun: the concept and study design, data collection, data analysis and interpretations, processing the draft of the manuscript, critical revision of the manuscript and article finalization.

## CONFLICT INTEREST STATEMENT

None of the authors have any conflicts of interest to declare.

## Data Availability

The data that support the findings of this study are available from the corresponding author upon reasonable request.

## References

[ijn13283-bib-0001] Allen, J. , Hutchinson, A. M. , Brown, R. , & Livingston, P. M. (2020). Evaluation of the TRANSITION tool to improve communication during older patients' care transitions: Healthcare practitioners' perspectives. Journal of Clinical Nursing, 29(13–14), 2275–2284. 10.1111/jocn.15236 32129530

[ijn13283-bib-0002] An, S. , Li, J. , Xie, W. , Yin, N. , Li, Y. , & Hu, Y. (2020). Extracorporeal shockwave treatment in knee osteoarthritis: Therapeutic effects and possible mechanism. Bioscience Reports, 40(11), BSR20200926. 10.1042/BSR20200926 33074309 PMC7670564

[ijn13283-bib-0003] Bilge, A. , Ulusoy, R. G. , Üstebay, S. , & Öztürk, Ö. (2018). Osteoarthritis. *Kafkas* . Journal of Medical Sciences, 8(1), 133–142. 10.5505/kjms.2016.82653

[ijn13283-bib-0004] Bilik, Ö. (2017). Preoperative and postoperative nursing care of the patients who underwent total knee replacement surgery. Turkiye Klinikleri Surgical Nursing ‐ Special Topics Journal, 3(1), 54–64.

[ijn13283-bib-0005] Çelik, M. , Taştan Çelik, S. , & Kayhan Tetik, B. (2021). Approach to knee osteoarthritis in the primary care with current guidelines. Ankara Medical Journal, 21(2), 304–316. 10.5505/amj.2021.15986

[ijn13283-bib-0006] Costa, M. F. B. N. A. D. , Sichieri, K. , Poveda, V. B. , Baptista, C. M. C. , & Aguado, P. C. (2020). Transitional care from hospital to home for older people: Implementation of best practices. Revista Brasileira de Enfermagem, 73(suppl 3), 1–8. 10.1590/0034-7167-2020-0187 33146267

[ijn13283-bib-0007] Doğan, N. , Göriş, S. , & Demir, H. (2016). Levels of pain and self‐efficacy of individuals with osteoarthritis. Pain, 28(1), 25–31. 10.5505/agri.2015.30085 27225609

[ijn13283-bib-0008] Gaffney, C. J. , Pelt, C. E. , Gililland, J. M. , & Peters, C. L. (2017). Perioperative pain management in hip and knee arthroplasty. Orthopedic Clinics of North America, 48(4), 407–419. 10.1016/j.ocl.2017.05.001 28870302

[ijn13283-bib-0009] Hanusch, B. C. , O'Connor, D. B. , Ions, P. , Scott, A. , & Gregg, P. J. (2014). Effects of psychological distress and perceptions of illness on recovery from total knee replacement. Bone Joint Journal, 96‐B(2), 210–216. 10.1302/0301-620X.96B2.31136 24493186

[ijn13283-bib-0010] Holm, I. , Pripp, A. H. , & Risberg, M. A. (2020). The active with osteoarthritis (AktivA) physiotherapy implementation model: A patient education, supervised exercise and self‐management program for patients with mild to moderate osteoarthritis of the knee or hip joint. A national register study with a two‐year follow‐up. Journal of Clinical Medicine, 9(10), 3112. 10.3390/jcm9103112 32993103 PMC7599935

[ijn13283-bib-0011] Kang, E. , Gillespie, B. M. , Tobiano, G. , & Chaboyer, W. (2019). General surgical patients' experience of hospital discharge education: A qualitative study. Journal of Clinical Nursing, 29(1–2), e1–e10. 10.1111/jocn.15057 31509311

[ijn13283-bib-0012] Korkmaz, N. , Coşkun, G. , & Boyraz, İ. (2022). Investigation of the relationship between kinesiophobia, pain, functional status and self‐efficacy in patients with knee osteoarthritis of different severity. Journal of Occupational Therapy and Rehabilitation, 10(1), 11–16. 10.30720/ered.906970

[ijn13283-bib-0013] Kranker, K. , Barterian, L. M. , Sarwar, R. , Peterson, G. G. , Gilman, B. , Blue, L. , Stewart, K. A. , Hoag, S. D. , Day, T. J. , & Moreno, L. (2018). Rural hospital transitional care program reduces Medicare spending. The American Journal of Managed Care, 24(5), 256–260. https://pubmed.ncbi.nlm.nih.gov/29851443/ 29851443

[ijn13283-bib-0014] Li, J. , Clouse, J. M. , Brock, J. , Davis, T. , Jack, B. , Levine, C. , Mays, G. P. , Mittman, B. , Nguyen, H. , Sorra, J. , Stromberg, A. , Du, G. , Dai, C. , Adu, A. , Vundi, N. , & Williams, M. V. (2022). Effects of different transitional care strategies on outcomes after hospital discharge—Trust matters, too. The Joint Commission Journal on Quality and Patient Safety, 48(1), 40–52. 10.1016/j.jcjq.2021.09.012 34764025

[ijn13283-bib-0015] Liu, Z. , Gao, L. , Zhang, W. , Wang, J. , Liu, R. , & Cao, B. (2020). Effects of a 4‐week Omaha System transitional care programme on rheumatoid arthritis patients' self‐efficacy, health status, and readmission in mainland China: A randomized controlled trial. International Journal of Nursing Practice, 26(4), e12817. 10.1111/ijn.12817 31985129

[ijn13283-bib-0032] Lorig, K. , Chastain, R. L. , Ung, E. , Shoor, S. , & Holman, H. R. (1989). Development and evaluation of a scale to measure perceived self‐efficacy in people with arthritis. Arthritis & Rheumatism: Official Journal of the American College of Rheumatology, 32(1), 37–44. 10.1002/anr.1780320107 2912463

[ijn13283-bib-0016] Lyu, Q. Y. , Huang, J. V. , Li, Y. X. , Chen, Q. L. , Yu, X. X. , Wang, J. L. , & Yang, Q. H. (2021). Effects of a nurse led web‐based transitional care program on the glycemic control and quality of life post hospital discharge in patients with type 2 diabetes: A randomized controlled trial. International Journal of Nursing Studies, 119, 103929. 10.1016/j.ijnurstu.2021.103929 33901941

[ijn13283-bib-0017] Magan, A. A. , Ahmed, S. S. , Paton, B. , Konan, S. , & Haddad, F. S. (2020). Does multimodal therapy influence functional outcome after total knee arthroplasty? Orthopedic Clinics of North America, 51(4), 453–459. 10.1016/j.ocl.2020.06.011 32950214

[ijn13283-bib-0018] Mardani, A. , Griffiths, P. , & Vaismoradi, M. (2020). The role of the nurse in the management of medicines during transitional care: A systematic review. Journal of Multidisciplinary Healthcare, 30(13), 1347–1361. 10.2147/JMDH.S276061 PMC7608001 33154651

[ijn13283-bib-0019] Morkisch, N. , Upegui‐Arango, L. D. , Cardona, M. I. , van den Heuvel, D. , Rimmele, M. , Sieber, C. C. , & Freiberger, E. (2020). Components of the transitional care model (TCM) to reduce readmission in geriatric patients: A systematic review. BMC Geriatrics, 20(1), 345–350. 10.1186/s12877-020-01747-w 32917145 PMC7488657

[ijn13283-bib-0020] Nall, R. W. , Herndon, B. B. , Mramba, L. K. , Vogel‐Anderson, K. , & Hagen, M. G. (2020). An interprofessional primary care‐based transition of care clinic to reduce hospital readmission. The American Journal of Medicine., 133(6), e260–e268. 10.1016/j.amjmed.2019.10.040 31877267

[ijn13283-bib-0021] NAON: National Association of Orthopaedic Nurses . (2021). Retrieved January 7, 2021, from Orthonurse.org website: http://www.orthonurse.org/

[ijn13283-bib-0022] Naylor, M. D. , Hirschman, K. B. , Toles, M. P. , Jarrín, O. F. , Shaid, E. , & Pauly, M. V. (2018). Adaptations of the evidence‐based transitional care model in the U.S. Social Science & Medicine, 213, 28–36. 10.1016/j.socscimed.2018.07.023 30055423

[ijn13283-bib-0023] Ohuabunwa, U. , Johnson, E. , Turner, J. , Jordan, Q. , Popoola, V. , & Flacker, J. (2021). An integrated model of care utilizing community health workers to promote safe transitions of care. Journal of the American Geriatrics Society, 69(9), 2638–2647. 10.1111/jgs.17325 34287819

[ijn13283-bib-0024] Patel, S. K. , Miller, A. , Chen, S. , Lindsay, A. , Gray, M. , & Su, Y. P. (2021). Transitional care management visits to improve coordination of care. The American Journal of Managed Care, 27(4), e130–e134. 10.37765/ajmc.2021.88622 33877780

[ijn13283-bib-0025] Pinto, P. R. , McIntyre, T. , Araújo‐Soares, V. , Costa, P. , & Almeida, A. (2015). Differential predictors of acute post‐surgical pain intensity after abdominal hysterectomy and major joint arthroplasty. Annals of Behavioral Medicine, 49(3), 384–397. 10.1007/s12160-014-9662-3 25288368

[ijn13283-bib-0026] Russell, T. G. , Buttrum, P. , Wootton, R. , & Jull, G. A. (2011). Internet‐based outpatient tele‐rehabilitation for patients following total knee arthroplasty. The Journal of Bone and Joint Surgery‐American, 93(2), 113–120. 10.2106/jbjs.i.01375 21248209

[ijn13283-bib-0027] Sun, J. N. , Chen, W. , Zhang, Y. , Zhang, Y. , Feng, S. , & Chen, X. Y. (2020). Does cognitive behavioral education reduce pain and improve joint function in patients after total knee arthroplasty? A randomized controlled trial. International Orthopaedics, 44(10), 2027–2035. 10.1007/s00264-020-04767-8 32772319

[ijn13283-bib-0028] Total Knee Replacement ‐ OrthoInfo ‐ AAOS . (2017). Retrieved January 3, 2021, from Aaos.org website: https://orthoinfo.aaos.org/en/treatment/total-knee-replacement/

[ijn13283-bib-0029] Tüzün, E. H. , Eker, L. , Aytar, A. , Daşkapan, A. , & Bayramoğlu, M. (2005). Acceptability, reliability, validity and responsiveness of the Turkish version of WOMAC osteoarthritis index. Osteoarthritis and Cartilage, 13(1), 28–33. 10.1016/j.joca.2004.10.010 15639634

[ijn13283-bib-0030] Ünsal, A. , & Kaşıkçı, M. (2008). Validity and reliability of self‐efficacy scale in arthritis. Atatürk Üniversitesi Hemşirelik Yüksekokulu Dergisi, 11(1), 40–50.

